# A pathologic study of Perivascular pTDP-43 Lin bodies in LATE-NC

**DOI:** 10.1186/s40478-024-01826-8

**Published:** 2024-07-12

**Authors:** Ryan K. Shahidehpour, Peter T. Nelson, Adam D. Bachstetter

**Affiliations:** 1 Spinal cord and brain injury research center, Sander-Brown Center on Aging, Department of Neuroscience, University of Kentucky, 741 S. Limestone St, Lexington, KY 40536 USA; 2 Department of Neuroscience, University of Kentucky, Lexington, KY USA; 3 Sanders-Brown Center on Aging, University of Kentucky, Lexington, KY USA; 4 Department of Pathology and Laboratory Medicine, Division of Neuropathology, University of Kentucky, Lexington, KY USA

**Keywords:** TDP-43, Alzheimer’s disease, LATE-NC, Astrocyte, Microglia, Iron

## Abstract

**Background:**

TAR DNA-Binding Protein 43 (TDP-43) pathological inclusions are a distinctive feature in dozens of neurodegenerative pathologies, including limbic-predominant age-related TDP-43 encephalopathy neuropathologic change (LATE-NC). Prior investigations identified vascular-associated TDP-43-positive micro-lesions, known as “Lin bodies,” located on or near the brain capillaries of some individuals with LATE-NC. This study aimed to investigate the relationship between the accumulation of Lin bodies and glial cells in LATE-NC and the potential co-localization with ferritin, a protein associated with iron storage. Using multiplexed immunohistochemistry and digital pathology tools, we conducted pathological analyses to investigate the relationship between Lin bodies and glial markers (GFAP for astrocytes, IBA1 for microglia) and ferritin. Analyses were conducted on post-mortem brain tissues collected from individuals with pathologically confirmed Alzheimer’s disease neuropathological changes (ADNC) and LATE-NC.

**Results:**

As shown previously, there was a robust association between Lin bodies and GFAP-positive astrocyte processes. Moreover, we also observed Lin bodies frequently co-localizing with ferritin, suggesting a potential link to compromised vascular integrity. Subsequent analyses demonstrated increased astrocytosis near Lin body-positive vessels compared to those without Lin bodies, particularly in ADNC cases. These results suggest that the accumulation of Lin bodies may elicit an increased glial response, particularly among astrocytes, possibly related to impaired vascular integrity.

**Conclusions:**

Lin bodies are associated with a local reactive glial response. The strong association of Lin bodies with ferritin suggests that the loss of vascular integrity may be either a cause or a consequence of the pTDP-43 pathology. The reactive glia surrounding the affected vessels could further compromise vascular function.

## Introduction

TAR DNA-Binding Protein 43 (TDP-43) pathological inclusions are pathologic features of more than 20 different neurodegenerative diseases, including limbic-predominant age-related TDP-43 encephalopathy (LATE-NC) and frontotemporal Lobar Degeneration with TDP-43 inclusions (FTLD-TDP) [1–3]. Whereas neuronal TDP-43 pathologies have been extensively studied, phosphorylated TDP-43 (pTDP-43) inclusions manifest in many different microenvironments, including those localized on or within small blood vessels, which are termed Lin bodies [4, 5]. These curious structures are approximately 5–10 μm in diameter and adherent to small vessels, particularly capillaries. Since the discovery of Lin bodies, other studies have briefly discussed vascular-associated TDP-43 in various diseases [5–7]. However, these studies likely included Lin bodies and other perivascular TDP-43 pathological inclusions.

To date, our understanding of Lin bodies remains limited. Immunohistochemical staining and immunoelectron microscopy indicate that Lin bodies are distinct from traditional pTDP-43 inclusions [4, 8]. They are associated with glial fibrillary acidic protein (GFAP)-positive astrocyte foot processes and, unlike other pTDP-43 pathologies, contain the B-crystallin heat shock protein [8]. While astrocytic endfeet envelop brain blood vessels and constitute a key part of the blood-brain barrier [9, 10], it is noteworthy that not all astrocytes express GFAP [7, 11]. Lin bodies near GFAP immunoreactivity may suggest a reactive astrocyte response to these bodies. This reaction could affect numerous astrocytic functions at the vasculature, potentially leading to loss of specific astrocytic endfoot protein polarization, vascular dysregulation, impaired waste clearance, and reduced vascular coverage. Alternatively, the association of Lin bodies with GFAP could be a coincidental occurrence due to increased reactive astrogliosis commonly observed in neurodegenerative conditions [12].

This project aimed to test the specificity of the association between Lin bodies and other glial structures. We hypothesized that if Lin bodies are specifically associated with GFAP immunoreactivity, there would be greater colocalization with GFAP than with the microglia marker IBA1 (ionized calcium-binding adaptor molecule 1), as both reactive microglia and astrocytes occur in greater numbers in tissue with neurodegenerative pathology. We further hypothesized that if Lin bodies contribute to microhemorrhage, this would correlate with increased erythrophagocytosis and subsequent ferritin expression, as iron from phagocytosed red blood cells is rapidly processed and stored as ferritin within cells [13]. Lastly, we investigated whether the association of GFAP, IBA1, or ferritin was more pronounced in capillaries associated with Lin bodies compared to capillaries without Lin bodies in the same individual and in capillaries from tissue with Alzheimer’s disease neuropathological changes (ADNC) without pTDP-43 pathology. The study results suggest that the localization of Lin bodies is relatively specific to GFAP and ferritin-immunoreactive microstructures.

## Methods

### Biospecimens

Formalin-fixed and paraffin-embedded human brain tissues, including the hippocampal formation, were obtained from the University of Kentucky Alzheimer’s Disease Research Center (UK-ADRC) biobank. Samples were selected based on the presence or absence of pTDP-43 pathology and ADNC. Summary information for each case is listed in Table 1.

### Immunohistochemistry

To assess GFAP immunoreactivity in proximity to TDP (+/-) vasculature, we utilized an adapted QUIVER protocol [14] to perform repeated staining on a single 10 μm tissue section from each case. Vascular structures were visualized using the CD34 antibody 1:200, Agilent, RRID: AB_2750581) and a permanent chromogenic substrate (ImmPACT^®^ SG Substrate Kit, Vector Laboratories, Cat #SK-4705). All subsequent rounds of cyclic multiplex immunohistochemistry utilized the removable ImmPACT AMEC Red Substrate kit (Vector Laboratories). The staining sequence for ensuing rounds included IBA1 (1:1000, Synaptic Systems, RRID: AB_2493179), ferritin (1:1000, Thermo Fisher, RRID: AB_259622), and GFAP (1:5000, Invitrogen, RRID: AB_2532994). After each staining round, a Zeiss Axio Scan Z.1 slide scanner was employed to capture and digitize complete slide images at 20x magnification with a 10 μm Z-stack comprised of 21 images taken every 0.5 μm and flattened into a single two-dimension image in Zen 2.6 Blue Edition. These images were then registered, deconvolved using the HALO deconvolution algorithm, and merged into a single pseudo-color image using the HALO Serial Registration module (Indica Labs, version 3.6).

### Identification and quantification of Lin bodies

Using the CD34 and TDP-43 channels, we identified vessels with Lin bodies (Lin body^+^ vessels), each approximately 8 μm in diameter, in the gray matter throughout all hippocampal subregions. Similarly, we randomly selected Lin body^−^ vessels in comparable brain regions in pTDP-43^+^ cases and pTDP-43^−^ cases. All vessels were at least 100 μm apart from other selected vascular structures. To ensure that our findings were associated to the presence of Lin body pathology and not an overall increase in gliosis and reactivity associated with disease, we quantified areas in close proximity to Lin bodies, while still capturing a representative sample of glia surrounding the Lin body^+^ vessel. Three concentric regions of interest were established for each vascular profile at 25 μm intervals. Two HALO image analysis macros were employed to quantify staining in these regions. The Area Fractionator algorithm quantified the overall staining burden, reporting results as the percentage of tissue covered by detected staining. The Object Colocalization algorithm counted the number of cellular profiles, with results reported as the number of cells per mm². For each marker, brightfield images were utilized to calibrate the Area Fractionator algorithm for automatic positive staining detection, using negative control samples to establish a baseline. The Object Colocalization algorithm was then applied to the original brightfield images to generate cell counts, with marker detection parameters based on stain intensity and size thresholds ranging from 50µm^2^ to 10,000µm^2^ to count only cells, excluding truncated processes. Finally, the Object Colocalization algorithm was used on the pseudo-color image to determine the co-occurrence of TDP-43^+^ Lin bodies with GFAP, IBA1, and ferritin. To increase fidelity and ensure accurate detection of Lin Body pathology, specifications within the HALO software were set to detect pTDP-43 structures between 10-100um^2^ in size. Additionally, in our tissue, we observed that Lin Bodies stained more intensely than other forms of pTDP-43 pathology, allowing us to further gate our detection method in HALO software based on an average staining intensity as well (< 0.2). After analyzing the piece of tissue in its entirety, each detected pTDP-43 structure was checked to ensure accuracy. Each Lin Body structure was assessed using only the pTDP-43 and CD34 staining channels, and the relative levels of IBA1, GFAP, or ferritin were unknown. Finally, we often observed instances of overlapping IBA1^+^ and GFAP^+^ signals. To confirm we were not capturing multiple cell processes laying on top of each other, all Lin Bodies found to colocalize with IBA1 and GFAP were omitted.

### Statistical analysis

Statistical analysis was conducted in GraphPad Prism (version 10.1). Glial reactivity was compared between TDP-43^+^ and TDP-43^−^ vessels within cases LATE-NC at each distance using paired T-tests. Comparison between LATE-NC and ADNC cases used unpaired T-test. The results are presented as mean ± SEM. Statistical significance was preset at an α level of 0.05.

## Results

Summary information about cases and controls is shown in Table 1. On average, individuals in the LATE-NC group were 10 years older, with an average age of death of 85.2 years, compared to 75.8 years in the pTDP-43^−^ ADNC group. While all cases in the ADNC group exhibited impaired cognitive function, one individual in the LATE-NC group did not have documented cognitive impairment. Additionally, the *APOE* ε4 allele was more common in pTDP-43^−^ cases than in pTDP-43^+^ cases. The male-to-female ratio was equal across both groups.

**Table 1:** Demographics of included cases from the University of Kentucky Alzheimer’s Disease Research Center (UK-ADRC) autopsy cohort. LATE-NC stage number was determined based on the latest consensus-based pathologic criteria [5]. The Thal phases is a method for scoring amyloid plaque distribution. Braak staging is used to score tau pathology distribution. Postmortem interval (PMI) is the time elapsed since the patient’s death and the autopsy, given in hours

Case	LATE-NC Stage	Thal Phase (Amyloid)	Braak Stage (Tau)	Clinical diagnosis	Age of death	Sex	PMI	APOE
**pTDP-43-positive cases**								
1	3	0	0	Demented	91	F	2.5	ε2/ε3
2	3	4	3	Normal	93	F	2.6	ε3/ε4
3	2	5	5	Demented	89	F	3.1	ε3/ε3
4	2	5	5	Demented	77	M	9.1	ε3/ε3
5	3	5	6	Demented	76	M	7.5	
**pTDP-43-negative cases**								
6	0	5	5	Demented	88	F	3.8	ε3/ε4
7	0	5	6	Demented	72	M	11.9	ε3/ε4
8	0	5	5	Demented	87	F	15.5	ε3/ε3
9	0	5	6	Demented	68	M	8.8	ε3/ε4
10	0	5	6	Demented	64	F	5.4	ε3/ε4

**Fig. 1:**
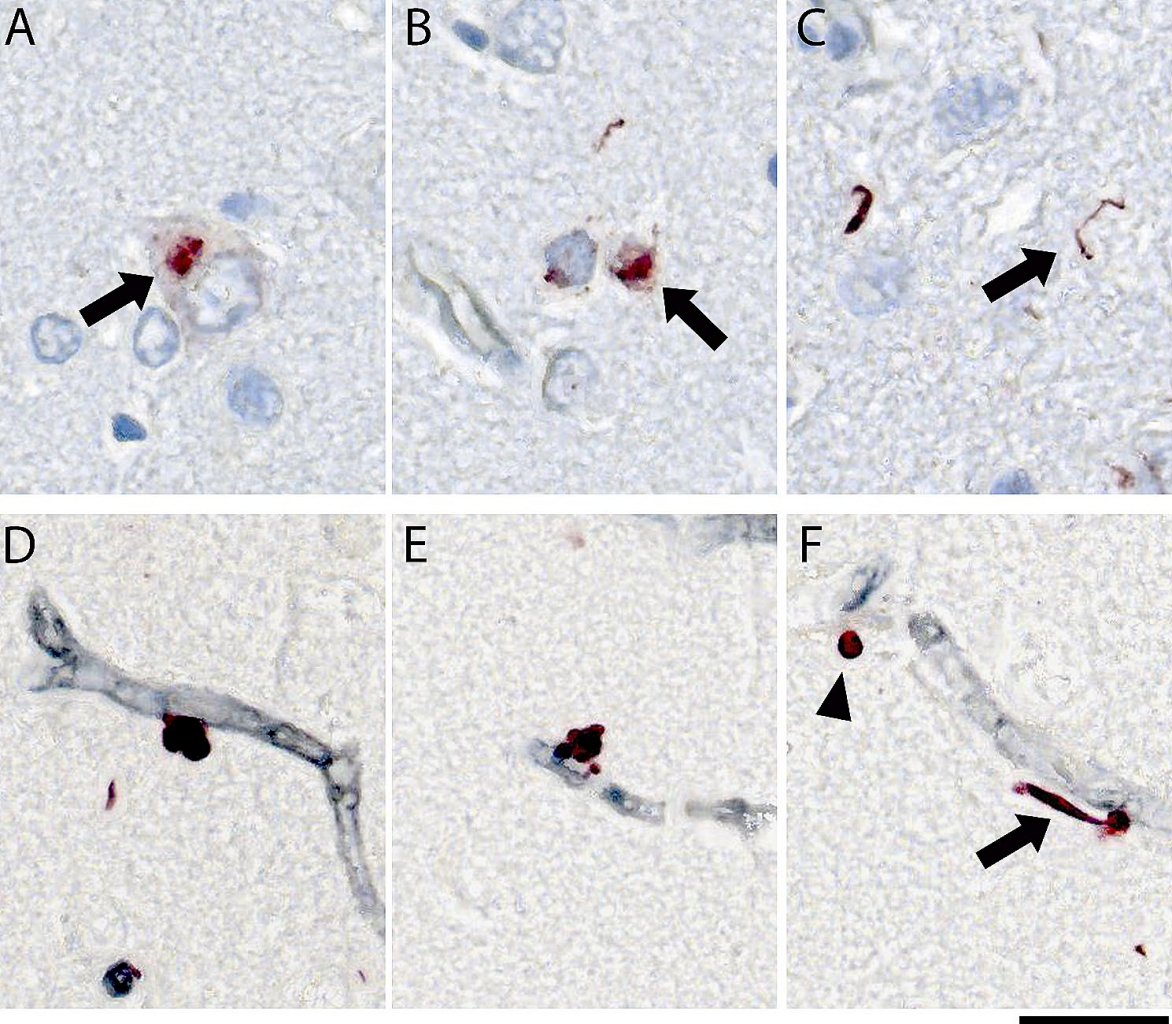
Illustration of the diverse pathological phenotypes of pTDP-43. Neuronal pTDP-43^+^ cytoplasmic inclusions **(A)**, intranuclear inclusions **(B)**, and dystrophic neurites **(C)** were commonly seen in LATE-NC cases. Distinct from these forms are Lin body-associated pTDP-43^+^ microvasculopathies. Using double chromogenic immunohistochemistry, which employs CD34 staining (black) to delineate vessels and pTDP-43 (red), the figure demonstrates multilobular doublet inclusions attached to the basal layer of the vascular space **(D)**. It also shows multilobular pTDP-43^+^ inclusions, with one lobe adhering to the vascular structure and the other lobes extending outward **(E)**. Additionally, single lobule inclusions are presented, which may occur with or without associated skein-like inclusions **(H)**, indicated by an arrowhead and an arrow, respectively. The scale bar indicates 25 μm in length

In its physiological state, TDP-43 is predominantly located within the nucleus. However, in pathological conditions, TDP-43 mislocalizes and forms deposits as cytoplasmic inclusions, as illustrated in Fig. 1A. It can also manifest as intranuclear inclusions (Fig. 1B) or elongated, thread-like inclusions comprising dystrophic neurites (Fig. 1C). TDP-43^+^ Lin bodies have been described in several TDP-proteinopathies, typically as multilobular doublet inclusions closely attached to the basal layer of the vascular space (Fig. 1D). Alternatively, they may present as multilobular inclusions, with one lobe adhering to the vascular structure and the other lobes extending outward (Fig. 1E). Instances of single lobule Lin body inclusions are also observed (Fig. 1F).

**Fig. 2:**
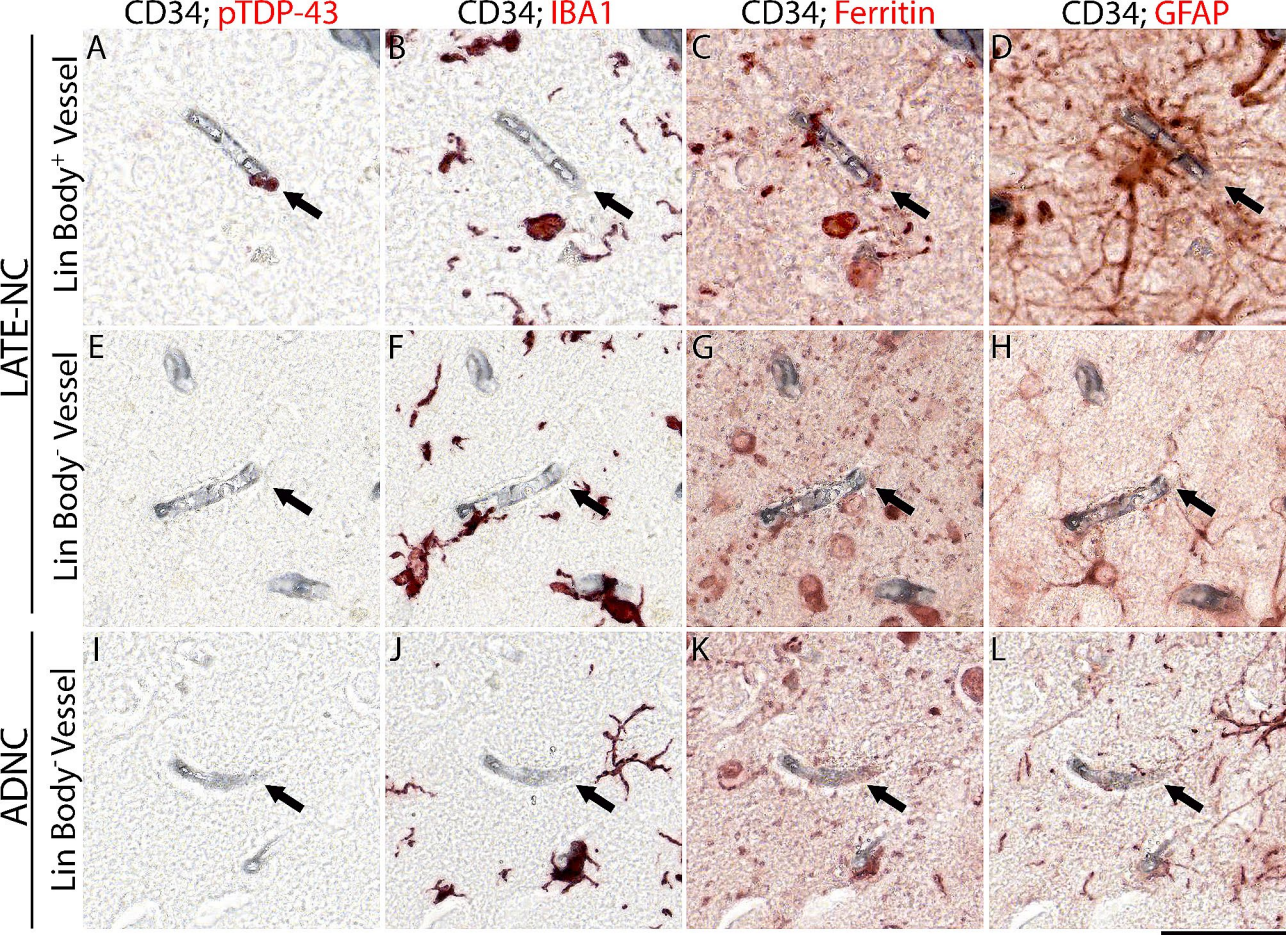
Multiplexed immunohistochemical staining to study glial cells near capillaries across various neurodegenerative diseases. The QUIVER method, employed on human formalin-fixed paraffin-embedded tissues, involves sequential multiplexed staining. Initially, the vasculature in pTDP-43^+^ and pTDP-43^−^ cases was stained for CD34 using a permanent chromogen. The sections were then stained for pTDP-43, highlighting vessels associated with Lin bodies and those without. This process was followed by consecutive staining rounds for IBA1, ferritin, and GFAP, each utilizing a removable chromogen. Arrows in the figure point to representative CD34-positive capillaries examined across different neurodegenerative diseases, illustrating the staining patterns observed for these markers in the respective disease states. The scale bar indicates 50 μm in length

**Fig. 3:**
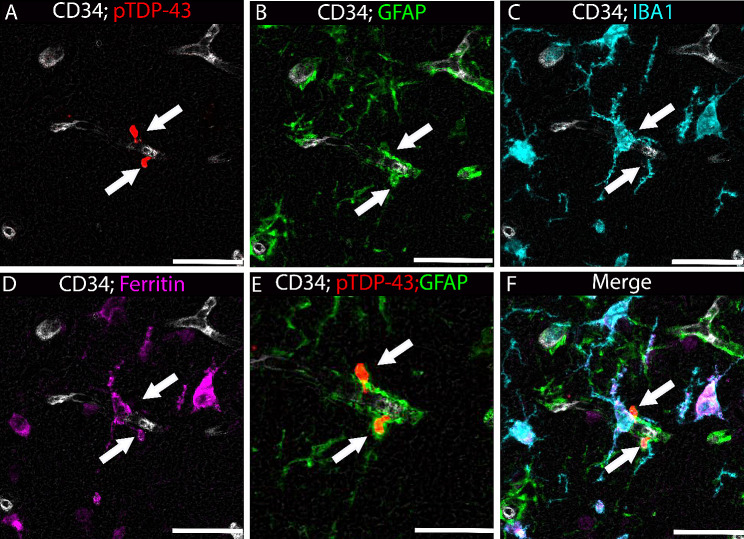
Pseudo-colored sequential immunohistochemistry of glia-associated and non-glia-associated Lin bodies. The representative images capture the presence of two Lin bodies apparently adherent to a single vascular profile. One Lin body is surrounded by GFAP and is also associated with ferritin. Although an IBA1-positive cell is near both Lin bodies, neither appears encapsulated within the microglia. A scale bar is included for reference, indicating a length of 25 μm

A technique integrating sequential multiple immunohistochemistry with a digital pathology tool was used to investigate the specificity of the association between Lin bodies and GFAP^+^ cells. This approach quantified the association of Lin bodies with GFAP, IBA1, and ferritin. Figure 2 shows the initial staining of tissues from pTDP-43^+^ and pTDP-43 cases with CD34, which labels vessels with a permanent black chromogen. Subsequently, the sections were stained sequentially for pTDP-43, GFAP, IBA1, and ferritin using the QUIVER method [14]. The CD34 staining ensured accurate co-registration of the serial stains, creating a pseudo-colored image shown in Fig. 3.

Qualitative analysis revealed frequent associations of Lin bodies with GFAP and ferritin. Cells positive for IBA1 and GFAP near Lin bodies often showed ferritin positivity. Consistent with prior reports, Lin bodies were commonly encircled by GFAP staining (Fig. 2) and found near microglia. While Fig. 3 does not depict this, some Lin bodies appeared internalized within microglial cells. In some instances, Lin bodies were not associated with GFAP or IBA1, as observable within the same capillary profile (Fig. 2). Notably, areas surrounding Lin body-positive vessels showed a significant presence of ferritin staining.

**Fig. 4:**
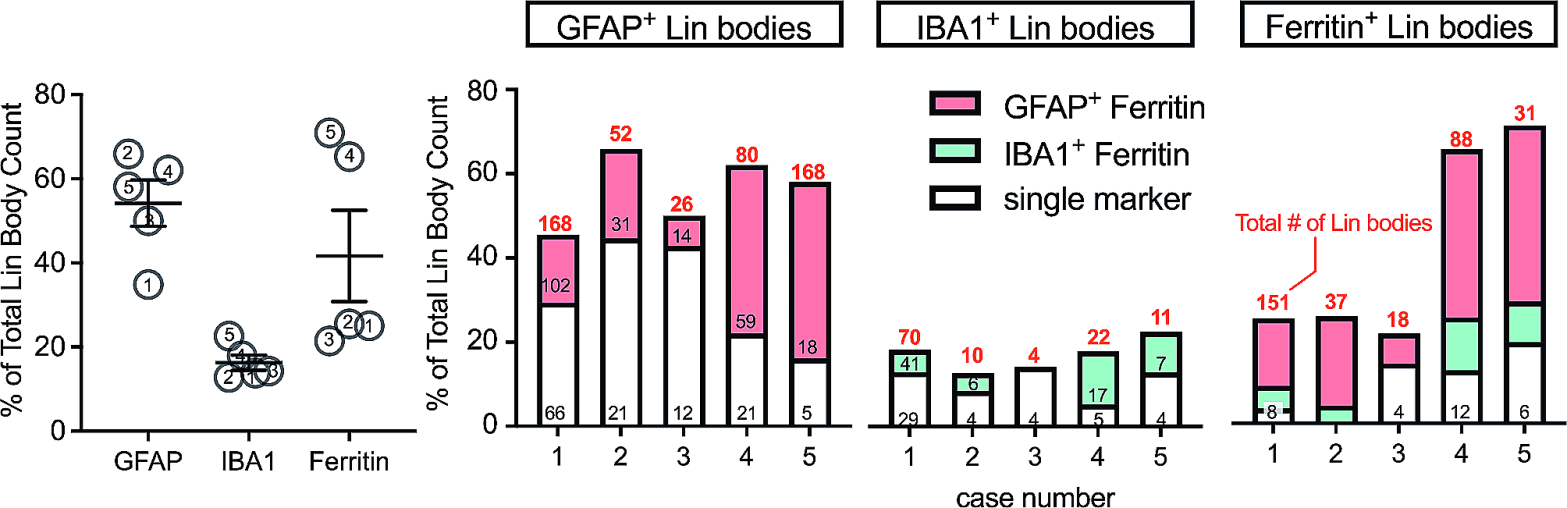
Lin body colocalization across ADNC^+/−^+LATE-NC cases. Despite the variation in the number of Lin bodies present per case, object colocalization of pTDP-43^+^ Lin bodies shows a greater association with GFAP and GFAP + ferritin than with IBA1 or ferritin alone. Numbers in each graph represent a single case (demographic information listed in Table 1). Numbers in red above each bar represent the total Lin body count immunoreactive for each marker, while the black number within each bar represents the Lin body count for each specific staining combination, i.e. (GFAP, GFAP + ferritin; IBA1, IBA1 + ferritin)

To quantify the association of pTDP-43^+^ Lin bodies with GFAP, IBA1, and ferritin, we used the object colocalization algorithm in HALO software, delineating a region of interest around each Lin body. Our analysis showed that 52% of Lin bodies were associated with GFAP staining, with nearly half of these (44%) also demonstrating colocalization with ferritin protein. Conversely, 18% of Lin bodies not colocalized with GFAP were found within IBA1 + microglia/macrophages. Approximately half of these microglia/macrophages containing Lin bodies also showed ferritin colocalization. Intriguingly, a minor percentage (7%) of Lin bodies did not appear to colocalize with IBA1 or GFAP but were associated with ferritin protein. Additionally, 20% of Lin bodies were not associated with GFAP, IBA1, or ferritin. Despite variability in the number of Lin bodies across different cases, this trend remained consistent in all analyzed cases, as illustrated in Fig. 4.

**Fig. 5:**
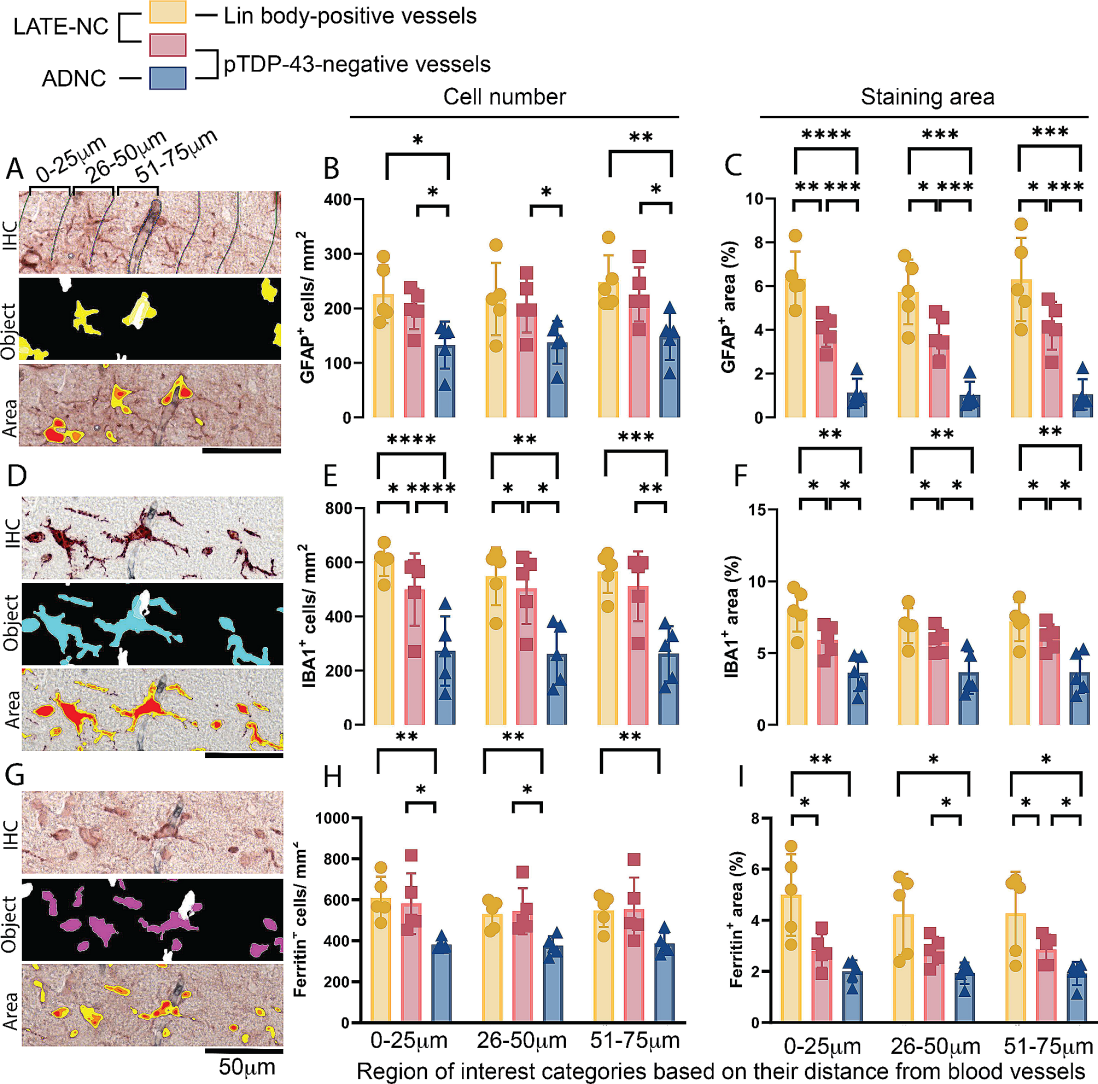
Specificity of glial responses to Lin bodies in neurodegenerative conditions. This figure compares the response of GFAP (**A-C**), IBA1 (**D-F**), and ferritin (**G-H**) in proximity to Lin body-positive vessels and pTDP-43-negative vessels of similar diameter, as shown in panel **A**. Panels A, D, and G illustrate automated tissue analysis markups for the Object Colocalization algorithm and the Area Fraction algorithm, showing what the software counted as cells and staining burden, respectively. The cell counts for GFAP, IBA1, and ferritin identified by this algorithm are displayed in panels **B**, **E**, and **H**, respectively. Within-case comparisons are made between LATE-NC Lin body-positive vessels (marked with yellow circles) and pTDP-43 negative vessels in the same LATE-NC cases (indicated by red squares). These are compared to pTDP-43 negative vessels in AD-NC cases (represented by blue triangles). Panels **C**, **F**, and **I** show the positively stained areas, as determined by the Area Fraction algorithm. Lin body-positive cases (indicated by yellow circles) exhibited a greater staining area than pTDP-43 negative vessels (red squares) within the same case for GFAP, IBA1, and ferritin (**C, F, I**). Vessels from pTDP-43 negative ADNC cases (blue triangles) showed the lowest levels of GFAP, IBA1, and ferritin in both cellular profiles (**B, E, H**) and staining area (**C, F, I**), compared to LATE-NC cases, regardless of ADNC pathology (AD +/-). Circles, squares, and triangles represent individual case results, with bars indicating the mean +/- SEM. Detailed statistical comparisons are provided in Table 2

While we observed a high association of GFAP with Lin bodies, we investigated further to determine if this association was non-specific. We compared GFAP’s association with Lin body-positive vessels to vessels without pTDP-43 pathology, both within the same case and in cases with high ADNC. Gliosis correlates strongly with neuropathology in LATE-NC and ADNC; we, therefore, hypothesized that a specific interaction between GFAP and Lin bodies would result in greater GFAP presence near Lin body-positive vessels than vessels without Lin bodies. Our findings revealed that, in Lin body-positive vessels, there was a significant increase in the area of GFAP staining but not in the number of GFAP cells (Fig. 5A-C). These findings suggest a localized GFAP increase in response to Lin bodies, while the overall number of GFAP cells remained indicative of the general neurodegenerative environment of the tissue.

In our further analysis, we examined the association of IBA1 with Lin body-positive vessels, as shown in Fig. 5E-F. Our automated cell count analysis of IBA1^+^ cells indicated a significant increase around capillaries in cases with pTDP-43 pathology compared to those without. Specifically, there was an increased number of IBA1^+^ cells within a 50 μm radius of vessels associated with Lin bodies. Beyond this radius, the increase in IBA1^+^ cells were present but not statistically significant, implying that these changes are most pronounced near the pathological sites.

Additionally, we assessed the average staining area for IBA1. The results mirrored the cell count findings, showing a larger staining area in pTDP-43-positive tissues than in pTDP-43-negative ones. Notably, vessels in LATE-NC cases showed a higher level of immunoreactivity than those in ADNC cases. These findings suggest that the presence of Lin bodies has a significant impact on the surrounding microenvironment, particularly in LATE-NC cases.

The impact of Lin bodies on vascular integrity and function remains uncertain. Considering ferritin’s role in safely processing and storing iron from phagocytosed red blood cells [13] and our findings of its frequent association with Lin bodies, we suggested a possible connection between Lin bodies and compromised vascular integrity. However, ferritin expression is also notably high in glial cells in age-related neurodegenerative diseases [14–16], leading us to investigate if ferritin expression was more pronounced in pTDP-43-positive vessels compared to pTDP-43-negative ones **(**Fig. 5G-I).

We observed the highest number of ferritin-positive cellular profiles in LATE-NC cases. However, there was no significant increase in ferritin-positive cells in Lin body-positive vessels compared to Lin body-negative vessels within these cases (Fig. 5H). Notably, the intensity of ferritin staining was greater near Lin bodies (Fig. 5I). These findings imply that while ferritin is commonly present in brains with neurodegenerative disease, especially in LATE-NC cases, its enhanced staining area around Lin body-positive vessels suggests a specific association with Lin bodies. These findings could indicate a response to local environmental changes, such as increased erythrophagocytosis, potentially occurring in microglia and astrocytes.

**Table 2:** Statistical association of glial responses to Lin bodies in neurodegenerative conditions. Bolded values indicate statistical significance (p < .05) for paired or unpaired T-tests, as specified in the table. ‘Distance’ refers to region of interest categories, classified by their proximity to blood vessels in micrometers (µm). ‘Number’ denotes cell count, and ‘Area’ represents the staining area, as determined by HALO software and illustrated in Fig. 5

		LATE-NC cases: Lin body vessels vs. pTDP-43 negative vessels (paired T-test)		pTDP-43 negative vessels: LATE-NC vs. ADNC (Unpaired T-test)		Lin body vessels vs. ADNC pTDP-43 negative (Unpaired T-test)	
	Distance	Number	Area	Number	Area	Number	Area
**IBA1**	0–25	**0.048**	**0.025**	**0.009**	**0.015**	**< 0.001**	**0.001**
	26–50	**0.029**	**0.025**	**0.028**	**0.021**	**0.006**	**0.005**
	51–75	0.088	**0.017**	**0.01**	**0.012**	**0.001**	**0.004**
**GFAP**	0–25	0.228	**0.007**	**0.032**	**< 0.001**	**0.016**	**< 0.001**
	26–50	0.403	**0.021**	**0.044**	**< 0.001**	0.051	**< 0.001**
	51–75	0.126	**0.017**	**0.032**	**< 0.001**	**0.001**	**< 0.001**
**Ferritin**	0–25	0.669	**0.012**	**0.019**	0.053	**0.002**	**0.004**
	26–50	0.734	0.051	**0.004**	**0.021**	**0.015**	**0.014**
	51–75	0.911	**0.042**	0.051	**0.021**	**0.006**	**0.014**

## Discussion

**Fig. 6:**
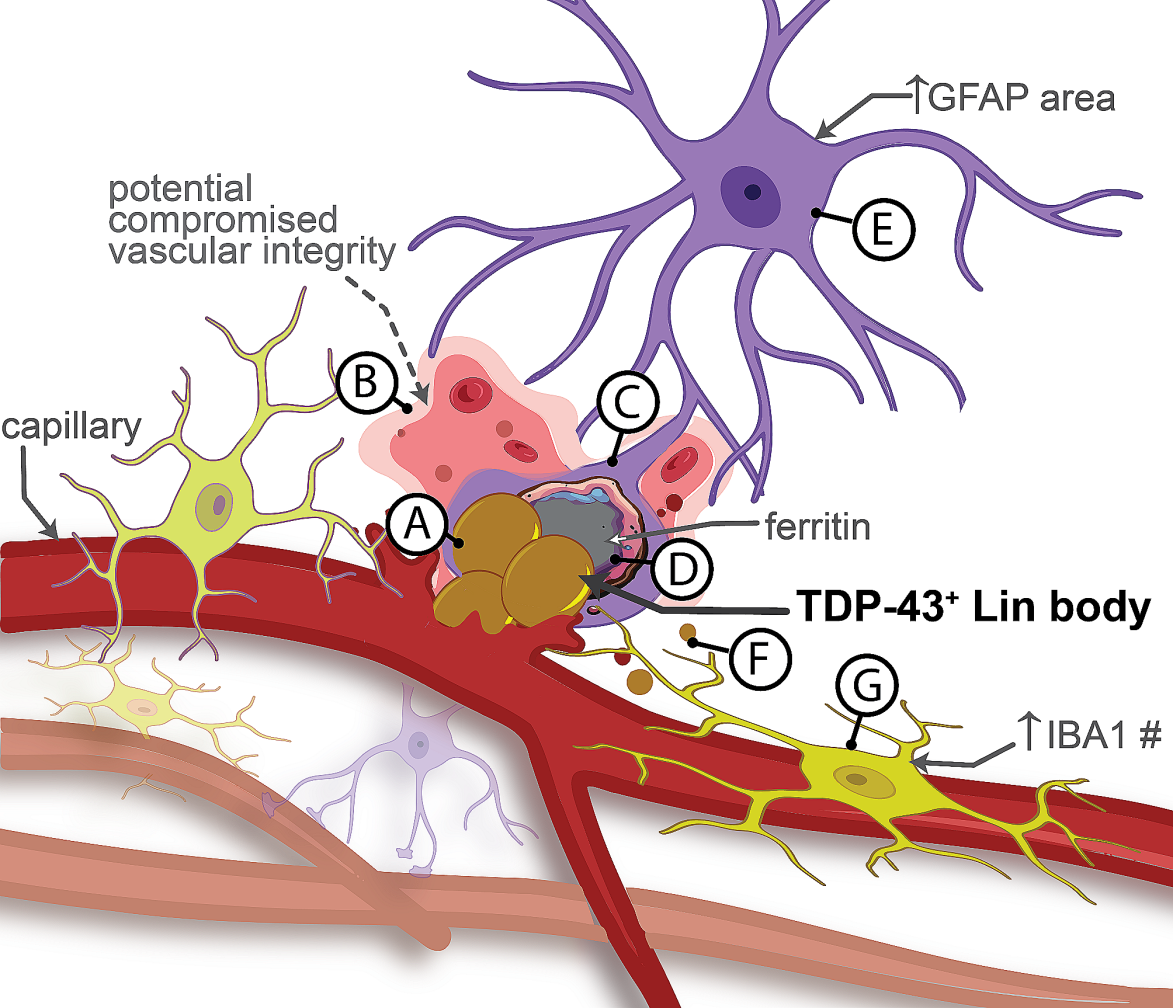
Proposed model for Lin Body interaction with glia. This figure illustrates our hypothesis for the potential sequala occurring with Lin Bodies and the resulting glial changes observed in and around capillaries throughout the brain. Vascular pathologies, such as arteriolosclerosis, occur in neurodegenerative diseases such as LATE-NC. However, it is now understood that TDP-43 within blood vessels is also associated with vascular changes, including breakdown and increased permeability of the BBB, resulting in TDP-43 leakage **(A)** and microhemorrhages **(B)**. In response, reactive astrocytes attempt to sequester leaking TDP-43, resulting in localization within the endfeet **(C)**. Following intracerebral hemorrhaging, processes such as erythrophagocytosis lead to increased intracellular levels of iron and iron storage proteins, including ferritin **(D)**. Once internalized, TDP-43 and increased levels of iron lead to astrocyte morphological changes without an apparent increase in the number of cells **(E)**. Typically, cerebral vasculature is covered by astrocytic endfeet, which may represent a first response to leakage of vascular TDP-43. Nonetheless, should astrocytes become incapable of an adequate response, we might observe an accumulation of perivascular Lin bodies not colocalizing with either GFAP or IBA1. **(F)**. Finally, microglia and peripheral macrophages are recruited to restore homeostasis, resulting in an increased population of IBA1^+^ cells surrounding pTDP-43^+^ vessels **(G)**

Cerebral vascular pathologies, such as arteriolosclerosis, are prevalent in LATE-NC cases [17–24]. Vascular pTDP-43 has recently been recognized as a distinct pathology termed a ‘Lin body’ (after the publication of Lin, 2009). There was a prior indication of a physical (proximity/colocalization) association between Lin bodies and GFAP, but the specificity of this relationship remained uncertain, particularly considering the proximity of astrocytic endfeet to blood vessels. Our findings provide further evidence that Lin bodies are specifically associated with GFAP/astrocytes, and this association is not merely coincidental, as evidenced by the reduced GFAP staining in similar vessels lacking pTDP-43 pathology (Fig. 6). Additionally, our study revealed a strong correlation between Lin bodies and ferritin, with ferritin staining intensifying near Lin body-positive vessels compared to others. The pronounced association between Lin bodies and ferritin might suggest the presence of loss of vascular integrity in areas of Lin bodies.

In a healthy brain, astrocytes have many functions, including maintaining the blood-brain barrier (BBB) and coordination of the cerebral blood flow. Morphological studies in post-mortem brains demonstrate close relationships between disease pathology and astrocyte reactivity [25, 26]. In humans, neuropathological changes associated with ADNC increase levels of astrogliosis, and the astrocyte reactivity is significantly worsened with comorbid pTDP-43 pathology as in ADNC + LATE-NC [12]. Our results agree with these findings and highlight regional increases in reactive astrocytes near pTDP-43 pathology.

Functional interactions of pTDP-43 pathology and astrocytes occur via various mechanisms [27, 28]. Experiments in rodent models have shown that alterations of non-phosphorylated TDP-43 may preferentially target hippocampal astrocytes and are sufficient to impair cell function and increase memory loss [29, 30]. Recently, astrocytes have also been highlighted as phagocytic cells within the CNS, demonstrating an ability to accumulate disease-associated pathology such as extranuclear TDP-43, making them a compelling area of interest in early-stage disease progression [25, 31, 32]. In a disease state, astrocytes are known to have decreased vascular coverage, in addition to increased reactivity and phagocytic activity [11, 25, 26, 33]. Furthermore, vascular TDP-43 pathology has recently been shown to increase BBB permeability [7, 34]. Therefore, one possible explanation for the presence of Lin bodies within astrocytic endfeet is that the breakdown of vasculature and increased BBB permeability allows for vascular pTDP-43 to leak out, where it is then phagocytized by glia, causing increased reactivity and inflammation within that cell.

Intracellular iron storage is largely carried out by the ferritin protein, which sequesters excess iron and stores it in a redox-inactive form for future use. Injuries such as traumatic brain injury, cerebral microbleeds, intracerebral hemorrhage, and neurodegenerative disease are associated with the rapid accumulation of red blood cells and iron in the brain parenchymal surrounding the injury, often leading to a secondary injury. In response to intracerebral hemorrhage, microglia, macrophages, and neutrophils phagocytose red blood cells in a process that is termed erythrophagocytosis [35–38]. When a cell is exposed to large concentrations of iron, such as following erythrophagocytosis, in-vitro studies suggest that their iron uptake rates temporarily exceed the storage capacity of their resident ferritin proteins, and the cells experience transient oxidative stress [39]. *In-vivo* animal model studies of iron uptake in astrocytes found that when activated via IL-1β and TNFα exposure, iron uptake increased three-fold compared to those at rest [39]. However, increased iron uptake observed in pathological conditions by both microglia and astrocytes leaves these cells in danger of heavy metal toxicity, leading glia to become triggers for further neurotoxicity associated with iron-induced oxidative stress and mitochondrial malfunctions [39, 40]. A prior study in the human brain showed colocalization of ferritin light chain protein with pTDP-43 in LATE-NC, but the specificity of the association was not assessed systematically [41].

In conclusion, our work provides new evidence of a qualitative feature of gliosis in human AD brains that is worsened with comorbid pTDP-43 pathology. Additionally, this work helps shed light on an intriguing subset of pTDP-43 pathology known as Lin Bodies that are observed on or within cerebral capillaries. Concurrent with previous findings, our digital pathology data shows a large population of these Lin bodies closely associated with GFAP^+^ cells, seemingly within astrocytic processes. Going beyond prior studies, we also found that many of these Lin Bodies colocalized with IBA1^+^ and ferritin protein-immunoreactive microstructures as well. These data may suggest that perivascular astrocytes are not only capable of phagocytizing pTDP-43 but do so at a higher rate than perivascular microglia/macrophages. Analyses of the glial response in close proximity to capillaries within the hippocampus of LATE-NC suggested that the presence of Lin bodies is sufficient to cause increased inflammation greater than what we see surrounding vessels without Lin bodies attached. Although more work is required to understand the role of vascular TDP-43 pathology, increased gliosis, and disease, this work helps us to elucidate the focal glial response elicited in various disease states because of TDP-43 microvasculopathies (Fig. 6).

## Data Availability

The datasets used and analyzed during the current study are available from the corresponding author upon reasonable request.

## Abbreviations

ADNC: Alzheimer’s Disease Neuropathological Changes
BBB: Blood-brain barrier
FTLD: TDP-Frontotemporal Lobar Degeneration with TDP-43
GFAP: Glial Fibrillary Acidic Protein
IBA1: Ionized Calcium Binding Adaptor Molecule 1
LATE: NC-TDP-43 Encephalopathy
TDP: 43-TAR DNA-Binding Protein 43
UK: ADRC-University of Kentucky Alzheimer’s Disease Research Center
